# The ViReflow pipeline enables user friendly large scale viral consensus genome reconstruction

**DOI:** 10.1038/s41598-022-09035-w

**Published:** 2022-03-24

**Authors:** Niema Moshiri, Kathleen M. Fisch, Amanda Birmingham, Peter DeHoff, Gene W. Yeo, Kristen Jepsen, Louise C. Laurent, Rob Knight

**Affiliations:** 1 Department of Computer Science & Engineering, University of California San Diego, La Jolla, CA USA; 2 Center for Computational Biology and Bioinformatics, University of California San Diego, La Jolla, CA USA; 3 Department of Obstetrics, Gynecology, and Reproductive Sciences, University of California San Diego, La Jolla, CA USA; 4 Department of Cellular and Molecular Medicine, University of California San Diego, La Jolla, CA USA; 5 Stem Cell Program, University of California San Diego, La Jolla, CA USA; 6 Institute for Genomic Medicine, University of California San Diego, La Jolla, CA USA; 7 Department of Pediatrics, University of California San Diego, La Jolla, CA USA; 8 Department of Bioengineering, University of California San Diego, La Jolla, CA USA; 9 Center for Microbiome Innovation, University of California San Diego, La Jolla, CA USA

**Keywords:** Data processing, Software

## Abstract

Throughout the COVID-19 pandemic, massive sequencing and data sharing efforts enabled the real-time surveillance of novel SARS-CoV-2 strains throughout the world, the results of which provided public health officials with actionable information to prevent the spread of the virus. However, with great sequencing comes great computation, and while cloud computing platforms bring high-performance computing directly into the hands of all who seek it, optimal design and configuration of a cloud compute cluster requires significant system administration expertise. We developed ViReflow, a user-friendly viral consensus sequence reconstruction pipeline enabling rapid analysis of viral sequence datasets leveraging Amazon Web Services (AWS) cloud compute resources and the Reflow system. ViReflow was developed specifically in response to the COVID-19 pandemic, but it is general to any viral pathogen. Importantly, when utilized with sufficient compute resources, ViReflow can trim, map, call variants, and call consensus sequences from amplicon sequence data from 1000 SARS-CoV-2 samples at 1000X depth in < 10 min, with no user intervention. ViReflow’s simplicity, flexibility, and scalability make it an ideal tool for viral molecular epidemiological efforts.

## Introduction

Molecular epidemiology uses viral genome sequences from patient samples to provide real-world public health insights about outbreaks^1^. Improved throughput of and access to sequencing technologies has dramatically increased viral sequence data production: one sequencing run on an Illumina NovaSeq S4 flow cell can yield raw viral sequence data from > 1500 patient samples^2^, and as of October 2021, over 4 million complete SARS-CoV-2 genomes have been deposited to the Global Initiative on Sharing All Influenza Data (GISAID) EpiCoV database^3^.

In a rapidly-growing pandemic, the time from raw sequence data to results (i.e., high-confidence variant calls and consensus sequences) is of utmost importance to implementing public health interventions in real-time. However, the sheer magnitude of raw viral sequence data that is collected poses a significant computational challenge. Many labs have access to sequencing technologies, but relatively few have experience with high-performance computing resources. Cloud computing platforms such as Amazon Web Services (AWS) are accessible and relatively inexpensive, but the optimal design and configuration of a cloud compute cluster typically requires systems administration expertise, and suboptimal cloud compute configuration can result in delays in time-to-results as well as in excess compute costs.

In this article, we present ViReflow, a user-friendly viral consensus sequence reconstruction and analysis pipeline enabling rapid analysis of large-scale viral sequence datasets using AWS and the Reflow system^4^. Reflow was chosen for its ability to automatically dynamically scale resource allocations on AWS without intervention from the user. To our knowledge, the only existing tools with similar functionality to ViReflow are V-pipe^5^, the nf-core/viralrecon pipeline^6^, HAVoC^7^, and ViralFlow^8^. A comprehensive pipeline comparison can be found in Table 1. In addition to being the only pipeline that supports viral lineage assignment^9^ beyond just Pangolin^10^ (via VirStrain^11^), the key benefits of ViReflow over the existing tools are its automatic cloud compute resource scaling for rapid cost-optimized parallel processing and its intuitive GUI. ViReflow’s simplicity and ease-of-use is critical to adoption by public health professionals who may have limited experience with command line interfaces.

## Methods

The ViReflow pipeline was built around Reflow, an incremental cloud-based data processing system developed by GRAIL (https://github.com/grailbio/reflow). ViReflow was developed specifically in response to the COVID-19 pandemic, but it is general to any viral pathogen. ViReflow implements the following standard viral consensus sequence workflow: (1) read trimming, (2) read mapping, (3) variant calling, and (4) consensus-sequence calling. ViReflow also implements optional analyses for specific viruses of interest, such as viral lineage calling (e.g. Pangolin for SARS-CoV-2). ViReflow extracts the core steps of our production pipeline (https://github.com/ucsd-ccbb/C-VIEW), implemented directly into AWS, which we have used to process tens of thousands of sequences in UC San Diego’s Return to Learn program (https://returntolearn.ucsd.edu)^12^. It packages these steps into a user-friendly tool that makes them accessible without ongoing user input or large-scale computational infrastructure, enabling rapid, scalable deployment across institutions.

**Table 1:** Pipeline comparison.

	V-pipe	nf-core/viralrecon	HAVoC	ViralFlow	ViReflow
Graphical user interface (GUI)	No	No	No	No	Yes
Amplicon sequencing support	No	Yes	Yes	Yes	Yes
Workflow tool	Snakemake^13^	Nextflow^14^	Bash script	Python script	Reflow
Native cloud compute support	None	AWS, GCP, Azure	None	None	AWS
Automatic compute resource scaling	No	No	No	No	Yes
Supported read trimmers	PRINSEQ^15^	Cutadapt^16^, fastp^17^, iVar^18^	fastp, Trimmomatic^19^	fastp	fastp, iVar, PRINSEQ, pTrimmer^20^
Supported read mappers	BWA-MEM^21^	Bowtie2^22^	Bowtie2, BWA-MEM	BWA-MEM	Bowtie2, BWA-MEM, HISAT2^23^, Minimap2^24^
Supported variant callers	LoFreq^25^	iVar, bcftools^26^	LoFreq	iVar	FreeBayes^27^, iVar, LoFreq
**Supported viral lineage assignment tools**	**None**	**Pangolin**	**Pangolin**	**Pangolin**	**Pangolin, VirStrain**
**Supported de novo genome assemblers**	**Haploclique**^28^**, SAVAGE**^29^**, ShoRAH**^30^	**minia**^31^**, SPAdes**^32^**, Unicycler**^33^	**None**	**None**	**MEGAHIT**^34^**, minia, SPAdes, Unicycler**

Bold denotes analyses that are optional in ViReflow.

**Figure 1:**
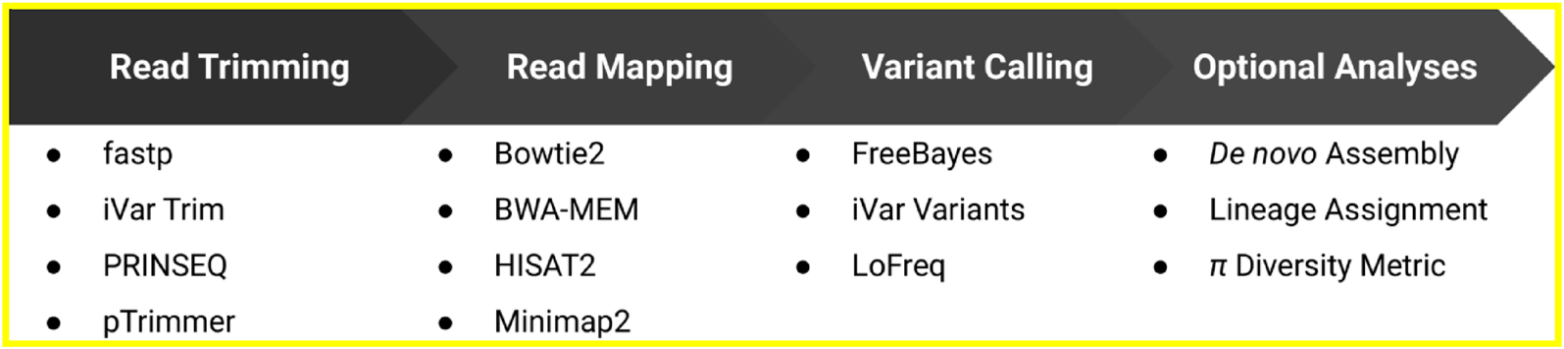
ViReflow pipeline. Vireflow implements a standard viral consensus sequence reconstruction pipeline, with multiple tool choices for each step of the pipeline. The output consensus sequence is produced by incorporating high-depth variant calls into the reference genome sequence.

ViReflow is modular: the user can choose amongst popular tools for each step (Fig. 1), and new tools will be added as they are developed. Importantly, when utilized with sufficient AWS resources, ViReflow’s overall runtime remains below 10 min even when processing one thousand samples. If the user experiences long runtimes due to high sequencing depth, the user can optionally provide an upper limit on the number of successfully-mapped reads (e.g. based on desired expected coverage), which speeds up read mapping and downstream analyses.

**Figure 2:**
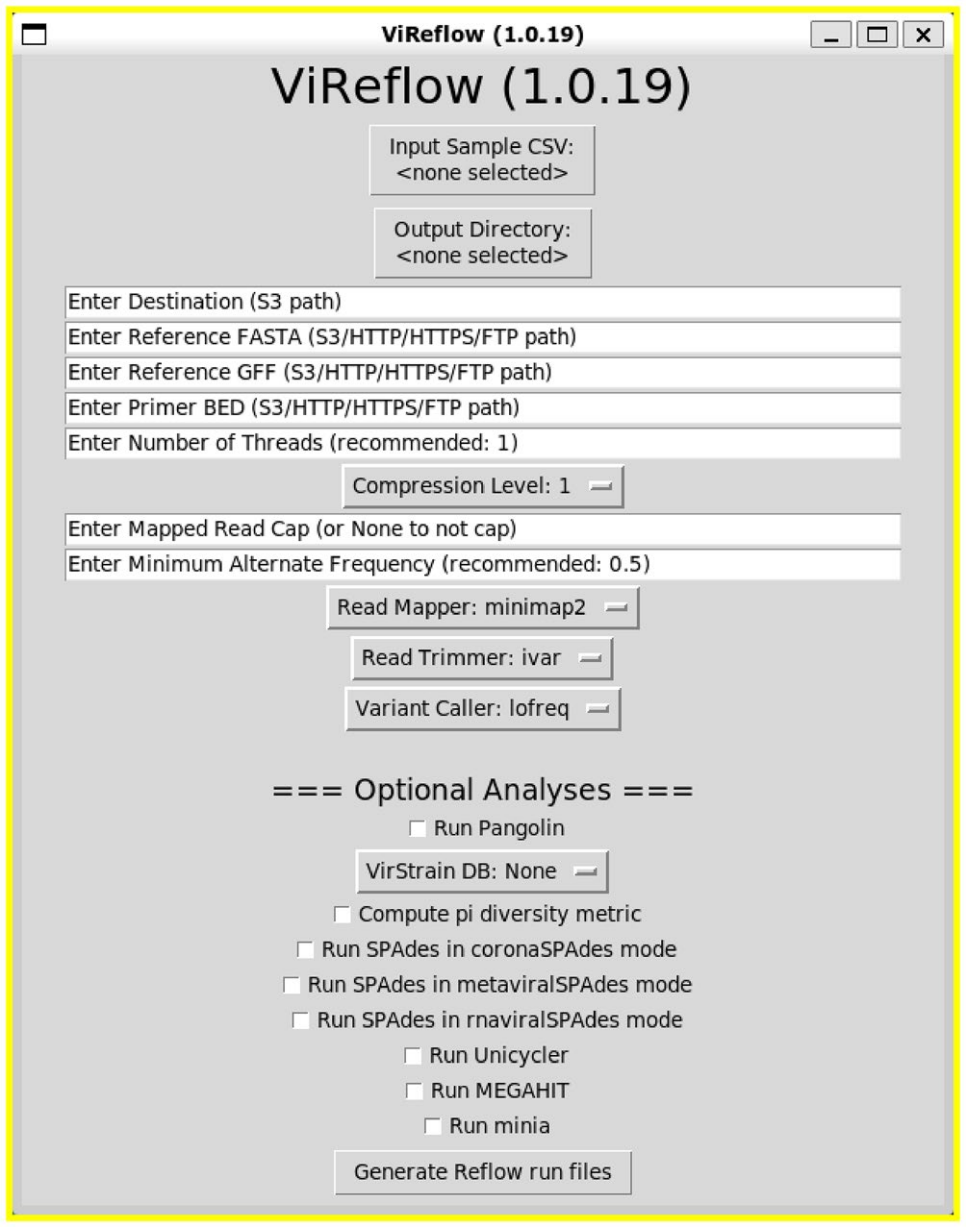
ViReflow Graphical User Interface (GUI).

Importantly, ViReflow is simple to install and run. The only ViReflow dependencies are Python (standard and cross-platform) and Reflow (distributed via Linux and Mac OS X binary), while ViReflow itself is just a single Python script that can be downloaded anywhere on the user’s machine. All other tool dependencies are configured automatically within AWS via pre-built minimal Docker containers without any intervention from the user, so the user need not install or configure any of the tools in the workflow. To run ViReflow, the user simply provides their AWS credentials as well as links to data and then executes ‘reflow run’ on the resulting Reflow runfile. The user can execute ViReflow from a command line interface as well as through a simple graphical user interface (GUI), which is implemented in native Python via Tkinter (Fig. 2).

Because ViReflow utilizes the Reflow runtime to execute the workflow, AWS compute resource allocations are automatically scaled based on each individual run’s needs, and Reflow attempts to execute all samples in a given run in parallel. Because the Reflow runtime supports AWS EC2 Spot Instances, ViReflow users can utilize unused EC2 capacity at significant discounts compared to the standard On-Demand instances (generally between 70 and 90% savings)^35^.

To enable reproducible research, each release version of ViReflow has a corresponding versioned Docker container. New ViReflow versions are released as new tools or features are added and as existing tools are upgraded. A Reflow runfile produced by ViReflow includes the specific ViReflow version and command that produced it, as well as the specific versioned ViReflow Docker container it used. Thus, if the user stores a runfile along with its corresponding FASTQ files, the complete analysis can be reproduced verbatim in the future.

To demonstrate ViReflow’s scalability, we benchmarked it using SARS-CoV-2 amplicon sequencing data produced using the SWIFT v2 protocol on an Illumina NovaSeq 6000. In brief, in one experiment, 342 biological samples were sequenced with paired end 150 basepair (PE150) reads across two lanes of an SP300 run to an average count of 2.85 million read pairs per sample. In a second experiment, 2,607 biological samples were sequenced PE150 across four lanes of an S4 flow cell to an average read count of 4.58 M read pairs per sample. We ran ViReflow in the default uncapped mode for the 342-sample run, and we ran ViReflow with a cap of 2 million successfully-mapped reads for the 2607-sample run. Due to library normalization issues, the sequencing depth varied considerably among samples, so the overall runtime (the maximum runtime across samples) was multiple hours long. In order to better study how ViReflow scales purely as a function of number of FASTQ pairs (*n*), we selected the single highest-depth sample from the 342-sample run and randomly subsampled its reads to produce FASTQ pairs with 1000 × depth (500× R1 and 500× R2 as matched pairs) *n* = 1, 10, 100, 1000, and 10,000 times to simulate multiple sequencing runs with the exact same sequencing depth. To account for stochasticity, we performed 10 technical replicates for each *n*, with the exception of *n* = 10,000, for which we only performed a single replicate due to cost constraints. We only allowed ViReflow to launch “standard” AWS EC2 instance types (A, C, D, H, I, M, R, T, and Z), capped at 96 vCPUs per instance. ViReflow v1.0.9 was executed in single-threaded mode (−t 1) using its default parameters. Our default AWS EC2 vCPU limit was too low to process datasets with over 100 samples, so we had to request increases in our vCPU and volume storage limits: to analyze *n* samples, we needed a vCPU limit of slightly more than *n* vCPUs and a volume storage limit of slightly more than 5*n* GB. FASTQ pairs were subsampled using seqtk^36^ v1.3. Runtimes were measured using the Linux ‘time’ utility, and total costs were obtained from AWS using Nutanix Beam. We utilized the NC_045512.2 SARS-CoV-2 reference genome and the SWIFT v2 primers. Our Reflow configuration file only allows “standard” AWS EC2 instance types (A, C, D, H, I, M, R, T, and Z).

To assess the quality of the consensus sequences produced by ViReflow, we turned to the ViralFlow manuscript, in which Dezordi et al.^8^ utilized a public dataset of 86 Brazilian SARS-CoV-2 Illumina paired-end amplicon sequencing libraries to compare the accuracy of consensus sequences produced by ViralFlow and HAVoC, and they demonstrated that ViralFlow had equal or improved accuracy with respect to HAVoC on all samples. We executed ViReflow v1.0.19 and ViralFlow v0.0.6, both single-threaded using their respective default parameters, on this exact dataset (EMBL-EBI study accession PRJEB47823). Due to the relatively low depth of the dataset, as per the ViralFlow documentation, both tools were run with a “minimum depth threshold” (for calling bases in the consensus sequence) of 5. To account for expected deviation in low-coverage regions at the ends of the genome, we compared consensus genome sequences between the start of ORF1a and the end of N with respect to the reference SARS-CoV-2 genome (i.e., positions 265–29,533).

## Results

In the benchmarking experiment, in the typical anticipated usage range for a sequencing run, 1 to 1000 samples at 1000 × depth per sample, given just raw untrimmed sequence data in FASTQ format, the total amount of time ViReflow required to perform read mapping, read trimming, variant calling, and consensus-sequence calling remained less than 10 min, and the total dollar cost scaled roughly linearly as a function of the total number of samples at approximately $0.005 per sample (Table 2). We note that it is also possible to run larger datasets that exceed the capacity of current sequencing technology: however, at 10,000 samples, the runtime jumped to ~ 3 h, and the dollar cost jumped to $0.12 per sample. Performance was excellent on real-world datasets: on the 342-sample NovaSeq run, ViReflow analyzed all 684 FASTQ pairs in under 2.5 h for $59.04 (approximately $0.086 per FASTQ pair), and on the 2,607-sample NovaSeq run, using a cap of 2 million successfully-mapped reads, ViReflow analyzed all samples in under 1.2 h for $117.48 (approximately $0.045 per sample).

**Table 2:** Benchmark of ViReflow.

# FASTQ pairs	Runtime (s)	Cost (USD)	Cost/Sample (USD)
1^S^	284 (4)	0.01 (2 × 10^–18^)	0.0100
10^S^	255 (12)	0.04 (0.003)	0.0041
100^S^	416 (21)	0.49 (0.024)	0.0049
1000^S^	491 (9)	5.65 (0.119)	0.0057
10,000^S^	12,075 (N/A)	1197.53 (N/A)	0.1198
684^R^	8,267 (N/A)	59.04 (N/A)	0.0863
2607^R,C^	4,144 (N/A)	117.48 (N/A)	0.0451

ViReflow was executed on 1, 10, 100, 1 K, and 10 K random 1000X depth sub-samplings of the single highest-depth sample from a NovaSeq SARS-CoV-2 amplicon sequencing run (denoted with^S^). ViReflow was also executed on two real NovaSeq runs (denoted with^R^), one of which was capped at 2 million successfully-mapped reads for each sample (denoted with^C^). All executions were run single-threaded. Total runtime (seconds) and total cost (US Dollars) across 10 technical replicates are shown as Mean (SD) pairs. “N/A” denotes single replicate execution due to high per-replicate compute costs. Specific details of tool choices (with versions) for each step of the pipeline can be found in the “Methods” section.

In the quality assessment experiment, in the region of the viral genome that was considered, ViReflow and ViralFlow produced identical consensus sequences on 67 of the 86 samples. For the remaining 19 samples, we manually inspected all differences between the pairs of consensus sequences in the context of their corresponding samtools depth and samtools mpileup results (to gauge the distribution of base calls and gaps in the trimmed BAM files at the corresponding positions)^37^. For all 19 discordant samples, samtools depth and samtools mpileup agreed with the ViReflow consensus sequence with respect to the chosen minimum depth and minimum alternate allele frequency parameters.

## Discussion

ViReflow is a user-friendly, scalable viral consensus sequence reconstruction tool that enables the rapid analysis of viral genomic sequencing data. ViReflow allows the user to select from multiple possible tools for each step of the pipeline, but without any need for system administration to configure those tools themselves. Importantly, in addition to its ability to scale automatically to support the analysis of ultra-large datasets, ViReflow produces genome consensus sequences that not only agree with existing pipelines, but which seem to potentially have slightly improved accuracy in specific cases when using ViReflow’s default settings, which were selected to provide a balance between accuracy and runtime.

We aimed to integrate as many best-practice tools as possible, and due to ViReflow’s modularity of tool selection, researchers can fine-tune their specific analyses as desired. Importantly, ViReflow can naturally evolve as improved tools for mapping and trimming reads, calling variants, and performing downstream analyses of interest (e.g. lineage assignment or abundance quantification) are developed.

ViReflow is available open source at https://github.com/niemasd/ViReflow, and it can be used to massively scale viral molecular surveillance efforts around the world by bringing high-performance cloud computing directly to public health officials and epidemiologists. After initial setup, instructions for which are thoroughly documented in the ViReflow repository, researchers can utilize a simple interface in order to execute a viral amplicon sequence analysis pipeline on tens, hundreds, or even thousands of samples without needing to worry about high-performance computing queues or cloud compute configuration.
